# Co-Infections in Children Hospitalised for Bronchiolitis: Role of Roomsharing

**DOI:** 10.4021/jocmr1556w

**Published:** 2013-10-12

**Authors:** Jolita Bekhof, Joline Bakker, Roelien Reimink, Mirjam Wessels, Veerle Langenhorst, Paul L.P. Brand, Gijs J.H.M. Ruijs

**Affiliations:** a Princess Amalia Children’s Clinic, Isala klinieken, Zwolle, The Netherlands; b Laboratory for Clinical Microbiology and Infectious Diseases, Isala klinieken, Zwolle, The Netherlands

**Keywords:** Respiratory syncytial virus, Isolation, Cross infections, Transmission, Nosocomial, Cohorting

## Abstract

**Background:**

Bronchiolitis is a major cause for hospitalisation in young children during the winter season, with respiratory syncytial virus (RSV) as the main causative virus. Apart from standard hygiene measures, cohorting of RSV-infected patients separately from RSV-negative patients is frequently applied to prevent cross-infection, although evidence to support this practice is lacking. The objective is to evaluate the risk of room sharing between RSV-positive and RSV-negative patients.

**Methods:**

We performed a prospective observational cohort study in children < 2 years hospitalised with acute bronchiolitis. During the first day of admission, patients shared one room, pending results of virological diagnosis (PCR). When diagnostic results were available, RSV-positive and RSV-negative patients were separated. Standard hygienic measures (gowns, gloves, masks, hand washing) were used in all patients.

**Results:**

We included 48 patients (83% RSV-positive). Co-infection was found in nine patients at admission, and two during hospitalisation (23%). The two patients with acquired co-infection had been nursed in a single room during the entire admission. None of 37 patients sharing a room with other bronchiolitis patients (20 with patients with a different virus) were co-infected during admission. Disease severity in co-infection was not worse than in mono-infection.

**Conclusion:**

One in five patients with bronchiolitis was co-infected, but co-infection acquired during admission was rare and was not associated with more severe disease. Room sharing between RSV-positive and RSV-negative patients (on the first day of admission) did not influence the risk of co-infection, suggesting that cohorting of RSV-infected patients separate from non-RSV-infected patients may not be indicated.

## Introduction

Acute bronchiolitis is a major cause for hospitalisation in young children during the winter season [1, 2]. Human Respiratory Syncytial Virus (RSV) is the most frequently identified virus, however with the use of new and highly sensitive molecular amplification methods, the role of other viral pathogens in bronchiolitis has been increasingly recognized. Various disease severity has been shown for a range of respiratory viruses, and double viral infection is relatively common, occurring in about 10-30% of hospitalised patients [3-7]. There is no consensus, however, on the impact of such co-infection on disease severity [5]: Some studies showed more severe disease in co-infected children [8-14], while others did not [15-21]. Most hospitals perform routine viral testing to identify and isolate RSV-infected infants, with the aim of reducing the risk of nosocomial cross-infection of other patients [22-24]. However, no good evidence is available of how effective this approach is in preventing nosocomial cross-infections among admitted patients with the clinical diagnosis of bronchiolitis.

Because of limited isolation facilities, patients with bronchiolitis admitted to our pediatric ward initially share a room, pending the results of virologicall diagnosis. We hypothesize that contact isolation measures and maintaining enough distance between the beds in a shared room should be sufficient in preventing cross-infection, since the major route of transmission of respiratory viruses is by close contact with infected secretions and not by small-particle aerosol [24, 25].

### Objectives

The purpose of this study was to determine the incidence of cross-infection in children hospitalised for bronchiolitis, when patients with RSV share the same room with patients with bronchiolitis infected with another virus during the first day of admission.

## Materials and Methods

The study was conducted at our 30-bed pediatric ward. From December 2011 through March 2012, all eligible infants younger than two years of age hospitalised for acute bronchiolitis were prospectively enrolled. Bronchiolitis was defined as acute respiratory disease, accompanied by coryza, cough, inspiratory crackles and/or expiratory wheezing on auscultation. Infants with chronic lung disease, congenital heart disease and Down’s syndrome were excluded.

We prospectively collected the following demographic and clinical information, including presence and number of room mates, virological diagnosis of the patient and room mates, and daily dyspnoea score assessed by an independent researcher, who was unaware of virological diagnosis (Table 1) [26].

**Table 1:** Dyspnoea Score

	0	1	2
Respiratory rate	normal < 40/min	slightly increased 40 - 60/min	clearly increased > 60/min
Oxygen saturation	≥ 95% in room air	92-94% in room air	< 92% in room air, or need for supplemental oxygen
Wheezing	none	audible with stethoscope	audible without stethoscope
Retractions	none	mild-moderate	severe
General condition	not affected: alert/quietly sleeping	moderately affected: Irritable or agitated	severely affected: lethargic, poor feeding

Adapted from Kristiansson [27].

A nasopharyngeal aspirate was collected for virological diagnosis by direct immunochromatographic antigen detection (RespiFinder TwoStep kit, Pathofinder) immediately at admission, every fourth day during admission, and five to seven days after discharge [27, 28].

All patients with bronchiolitis were treated with standard hygienic measures. Medical and nursing personnel wore gowns, gloves and masks during patient contact and washed their hands before and after patient contact. Parents and visitors were asked to wash hands before leaving the room. On the first day of admission, pending the results of the RSV-PCR, patients shared a two- or four-bed room, with beds separated at least 1.5 meter. Cohorting of RSV-infected patients commenced as soon as the result of RSV-PCR was known, generally within one day after admission.

### Statistical analysis

Chi-square test was used to compare categorical data, Mann-Whitney U-tests for continuous data because of skewed distributions. Statistical analyses were performed using Statistical Package for the Social Sciences (SPSS) version 19.

This study is registered at clinicaltrials.gov (NCT01441466).

## Results

Of the 84 patients with bronchiolitis hospitalised during the 11-week study period, 36 were excluded for the following reasons: cardiac disease (2), chronic lung disease with home oxygen (2), Down’s syndrome (3), no parental consent (12), age > two (7), missed inclusion (6), missing nasal wash specimen at admission (3). A total of 48 patients completed the study (Table 2).

**Table 2:** Patient Characteristics

n = 48	
Age, months	3.2 (1.8 - 9.7)
Male	26 (54.2%)
Birth characteristics	
gestational age, weeks	38.5 (37.8 - 40.1)
preterm birth (< 37 weeks)	2 (4.2%)
birth weight, gram	3420 (3,120 - 3,740)
Environmental factors	
day care attendance	16 (33.3%)
siblings	39 (81.2%)
Disease severity	
length of hospitalization (days)	1.9 (1.6 - 4.0)
oxygen supplementation	30 (62.5%)
tubefeeding	20 (41.7%)
highest dyspnoea score (0-10)	3.0 (2.0 - 4.8)
mechanical ventilation	3* (6.2%)

Data are presented as median and interquartile range in parentheses, or number and percentage in parentheses; Highest possible dyspnoea score 10; * all 3 patients mono-infected with RSV.

The distribution of viral pathogens is shown in Table 3; RSV was the major pathogen detected in 83%. Co-infection was found in 11 (22.9%) patients, nine of whom were already co-infected at admission, and two acquired co-infection during admission.

**Table 3:** Distribution of Viral Pathogens

Virus	At admission n = 48	At discharge n = 48	After discharge n = 44
Mono-infections			
RSV-A	7 (14.5%)	6 (12.5%)	2 (4.5%)
RSV-B	25 (52.1%)	19 (39.6%)	5 (11.4%)
hMPV	2 (4.2%)	1 (2.1%)	1 (2.3%)
RhV	3 (6.3%)	3 (.3%)	3 (6.8%)
CoV	0	2 (4.2%)	1 (2.3%)
AdV	0	0	3 (6.8%)
Co-infections			
RSV-A and hMPV	1 (2.1%)	1 (2.1%)	0
RSV-B and			
PIV	1 (2.1%)	0	0
AdV	0	1 (2.1%)	0
RhV	4 (8.3%)	4 (8.3%)	1 (2.3%)
CoV	1 (2.1%)	0	0
hMPV	1 (2.1%)	1 (2.1%)	0
CoV and PIV	1 (2.1%)	1 (2.1%)	0
No virus	2 (4.2%)	9 (18.8%)	28 (63.6%)

Number with percentage in parentheses; RSV: Respiratory Syncytial; hMPV: human MetaPneumo Virus; RhV: Rhino Virus; CoV: Corona Virus; AdV: Adeno Virus; PIV: Parainfluenza Virus.

Of all included patients, 37 (77.1%) had shared a room with other bronchiolitis patients, 20 of whom (54.1%) had shared a room with a patient infected with a different virus. The two patients who acquired co-infection during admission had never shared a room with another patient. None of the bronchiolitis-patients sharing rooms had been infected with another virus during admission.

Co-infected patients did not suffer from more severe disease than patients infected with a single virus, but, although not statistically significant, disease severity tended to be higher in RSV-infected patients compared to RSV-negative patients (Table 4).

**Table 4:** Comparison of Disease Severity Between Mono- Versus Co-Infected Patients and RSV-Infected Versus RSV-Uninfected Patients

n = 48	Mono versus co-infection			RSV-infected versus RSV-uninfected		
	Co-infection n = 11	Mono-infection n = 37	P-value	RSV-infected n = 40	RSV-uninfected n = 8	P-value
Age, months	4.3 (2.2 - 11.4)	3.2 (1.6 - 9.4)	0.413	3.3 (1.8 - 9.8)	3.0 (1.6 - 8.5)	0.740
Length of hospitalization, days	2.0 (1.7 - 3.4)	1.9 (1.2 - 4.2)	0.864	2.5 (1.6 - 4.4)	1.8 (1.2 - 1.9)	0.162
Oxygen supplementation	6 (54.6%)	24 (64.9%)	0.535	25 (62.5%)	5 (62.5%)	1.000
Tubefeeding	4 (36.4%)	16 (43.2%)	0.681	18 (45.0%)	2 (25%)	0.295
Highest dyspnoea score (0-10)	3.0 (2.0 - 4.0)	3.0 (1.5 - 5.0)	0.654	3.0 (2.0 - 5.0)	2.5 (1.0 - 4.0)	0.285
Mechanical ventilation	1 (9.1%)	2 (5.4%)	0.658	3 (7.5%)	0 (0%)	0.424

Data are presented as median and interquartile range in parentheses, or number and percentage in parentheses as appropriate; P value: Mann-Whitney-U test for continuous variables, χ^2^ test for dichotomous variables.

## Discussion

This study showed that nosocomially acquired co-infection is rare, even when RSV-positive and RSV-negative patients share a room during the first day of hospital admission. Furthermore, co-infection was not associated with more severe disease. The small number of our study limits any firm conclusion, however these findings may suggest that separating RSV-infected from RSV-negative patients with bronchiolitis may not be indicated. Cohorting of patients with bronchiolitis as one group, irrespective of viral diagnosis, may suffice.

Our finding that cohorting of RSV-infected patients may not add to the prevention of co-infection is supported by the fact that the main route of transmission of respiratory viruses is through direct contact, with only a minor role for aerosol transmission [24, 25]. Therefore, we stress that strict adherence to other hygienic measures by medical staff and patient’s relatives is clearly of crucial importance [23, 24]. Hand washing is the single most important procedure in the prevention of nosocomial infections, yet it remains the most violated of all infection control procedures [23, 24]. It is conceivable that placing children in a cohort generates considerable peer and parental pressure to ensure that measures such as hand washing are followed.

Our results may also imply that routinely performing virological diagnostic testing is not needed in children with bronchiolitis. The diagnosis of bronchiolitis is a clinical diagnosis and for this purpose further diagnostic testing is not needed [29]. Since cohorting of RSV-infected patients is the most importance reason for virological testing in bronchiolitis, health care expenses can be reduced by omitting the routine use of these tests, provided that influenza, a serious and treatable infection, is excluded.

This does not exclude the potential usefulness of rapid broad range viral testing in specific circumstances, for example in young febrile infants, where rapid broad range viral testing might reduce the need for invasive sepsis workup, or in case of unclear clinical presentation (apnoea without respiratory signs) or for surveillance purposes.

Our findings add to the current controversy considering this issue and we realise that the small numbers of our study limit solid comments on this subject and no definite conclusions can be made. Another important limitation is the fact that we only evaluated the risk of room sharing during the first 24 hours of admission. It is well possible that prolonged sharing of rooms increases the incidence of cross-infections. For practical and safety reasons, we deliberately chose to perform the study under these specific circumstances as a proof of principle, before embarking on a similar project with room sharing during the entire admission.

We conclude that, with standard hygiene control measures, the risk of nosocomially acquired co-infection is low, and does not appear to be related to room sharing between RSV-positive and RSV-negative patients (during the first day of admission). These findings argue against routine cohorting of RSV-infected bronchiolitis patients and against routinely carrying out broad range virological testing of infants hospitalised for bronchiolitis. Yet a larger number of patients, applying room sharing during the entire admission is needed before definite conclusions can be made.
